# Circulating Circular RNAs: Novel Biomarkers for Heart Failure

**DOI:** 10.3389/fphar.2020.560537

**Published:** 2020-11-13

**Authors:** Chuan Sun, Mingming Ni, Bo Song, Lu Cao

**Affiliations:** Department of Pharmacy, Children’s Hospital of Nanjing Medical University, Nanjing, China

**Keywords:** heart failure, circRNAs, diagnosis biomarkers, exosome, non-coding RNA

## Abstract

Heart failure (HF) is a serious, chronic disease, causing significant ill health and high mortality worldwide. The current clinical strategies emphasize reducing the transition from a healthy to a failing heart despite the shift in the clinical goal from healing to disease prevention. Recent research advancements on noncoding RNAs (ncRNAs) have demonstrated that circular RNAs (circRNAs) are significant therapeutic targets in HF. Previous studies have highlighted the potential applicability of circRNAs in the diagnosis and treatment of diseases. However, less is known regarding the potential benefits of circRNAs as novel diagnostic and treatment biomarkers for HF. In the present study, we summarize the current developments and achievements associated with the use of circRNAs as HF biomarkers. We also discuss future research directions regarding HF diagnosis and treatment.

## Introduction

Heart failure (HF) is attributed to several cardiovascular diseases (CVDs), including, cardiac hypertrophy, myocardial fibrosis, and ischemic cardiomyopathy. In 2000, the prevalence of chronic heart failure (CHF) in the Chinese population aged 35–74 years was 0.9%, and CHF prevalence was found to significantly increase with age. The China Heart Failure Patient Registration Research (China-HF) analyzed data from 8516 patients with HF admitted in 88 participating hospitals from 2012 to 2014 and reported a significant increase in the average age of patients hospitalized with HF. Hypertension and coronary heart disease are reported as the main causes of HF in China, and infections have been implicated in the pathogenesis of HF. Currently, the mortality rate after hospitalization with HF is 4.1% in China from 2014 to 2018, which is significantly lower than the rate in previous years. Patients with HF present with nonspecific symptoms and are difficult to distinguish from those of other cardiovascular diseases. Some of the symptoms presented by patients with HF include dyspnea, orthopnea, and paroxysmal nocturnal dyspnea. In addition, the lack of adequate cardiac output can cause fatigue, weakness, and exercise intolerance. The clinical diagnosis of HF based on clinical manifestations is challenging and may lead to a definitive diagnosis and delayed treatment, thereby causing inaccurate prognosis (Gaggin and Januzzi, 2013).

Consequently, accurate and effective diagnostic measures are needed in clinical practice to distinguish HF from other cardiovascular diseases. Currently, medical history, physical examination, and chest X-rays are the traditional clinical assessment approaches for patients suspected to have HF. However, isolated signs and symptoms cannot be objectively assessed for HF diagnosis (Di Marca et al., 2018). With recent advances in medical technology, noninvasive imaging techniques, such as echocardiography and radionuclide angiography are being used to determine ventricular ejection fraction, diastolic function, and chamber pressures that may help diagnose HF. HF can be categorized as either asymptomatic or congestive HF. Patients with asymptomatic HF have ventricular diastolic dysfunction and abnormal ejection fraction with no accompanying clinical symptoms (Naji et al., 2017), further complicating HF diagnosis. Although the use of both noninvasive and invasive methods to examine HF is relatively accurate, patients with abnormal laboratory examinations may not present HF symptoms as previously demonstrated, which may confuse patients. Therefore, the development of novel biomarkers can predict and diagnose HF, risk stratification of patients with HF, and even serve as therapeutic targets are of biological significance (Gaggin and Januzzi, 2013).

## Limitations of Clinically Used Biomarkers

B-type natriuretic peptide (BNP) and N-terminal proBNP (NT-proBNP) have been widely used as diagnostic biomarkers for HF (Cao et al., 2019). In addition, the American Heart Association/American College of Cardiology (AHA/ACC) and European Society of Cardiology (ESC) guidelines recommend the use of BNP and NT-proBNP for clinical diagnosis of HF (Ponikowski et al., 2016; Yancy et al., 2017). However, diagnosing HF only depending on the concentrations of BNP and NT-proBNP are not accurate enough. Numerous factors can influence the concentrations of BNP and NT-proBNP (Nevo et al., 2011). For example, renal failure affects the metabolism of the two biomarkers and then increases their concentration (Medvedeva et al., 2017). In addition, BNP and NT-proBNP may be influenced by low specificity and fluctuations in circulation levels, including obesity, pulmonary embolism, patients’ age, and sex (Metra and Teerlink, 2017; Salgado-Somoza et al., 2017). Therefore, AHA/ACC and ESC guidelines do not recommend biomarker-guided therapy in the management of HF patients (Scali et al., 2014). Combining the concentrations of BNP and NT-proBNP with clinical syndrome, bio-humoral (such as renal function), and echocardiographic assessment may be much more accurate, but it also would delay the diagnosis of HF.

## The Potential Roles of miRNAs and lncRNAs as Biomarkers of HF

**Figure 1:**
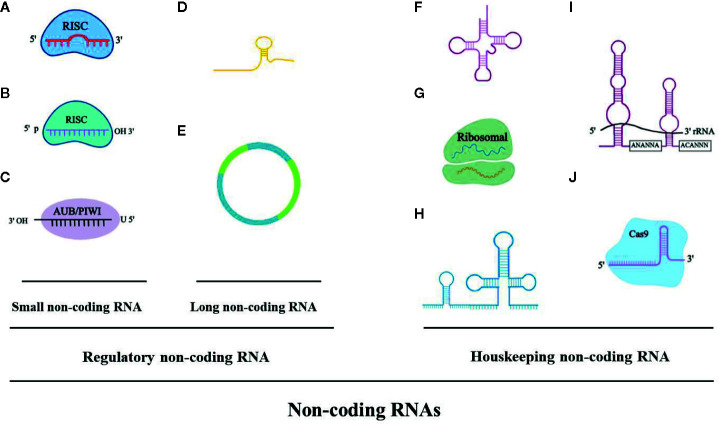
The noncoding RNA family. **(A)** microRNAA: miRNA, 19-23 bp. **(B)** Piwi-interacting RNA: piRNA, 24-30 bp. **(C)** Small interfering RNA: siRNA, 21-25 bp. **(D)** Long noncoding RNA: lncRNA, > 200 bp, linear. **(E)** Circular RNA: circRNA, > 200 bp, circular. **(F)** Transfer RNA: tRNA, 74-95 bp. **(G)** Ribosomal RNA: rRNA, 121-5000 bp. **(H)** Small nuclear RNA: snRNA, 100-300 bp. **(I)** Small nucleolar RNA: snoRNA, 100-300 bp (i.e. H/ACA box snoRNA). **(J)** Guide RNA: gRNA, 55-70 bp.

**Figure 2:**
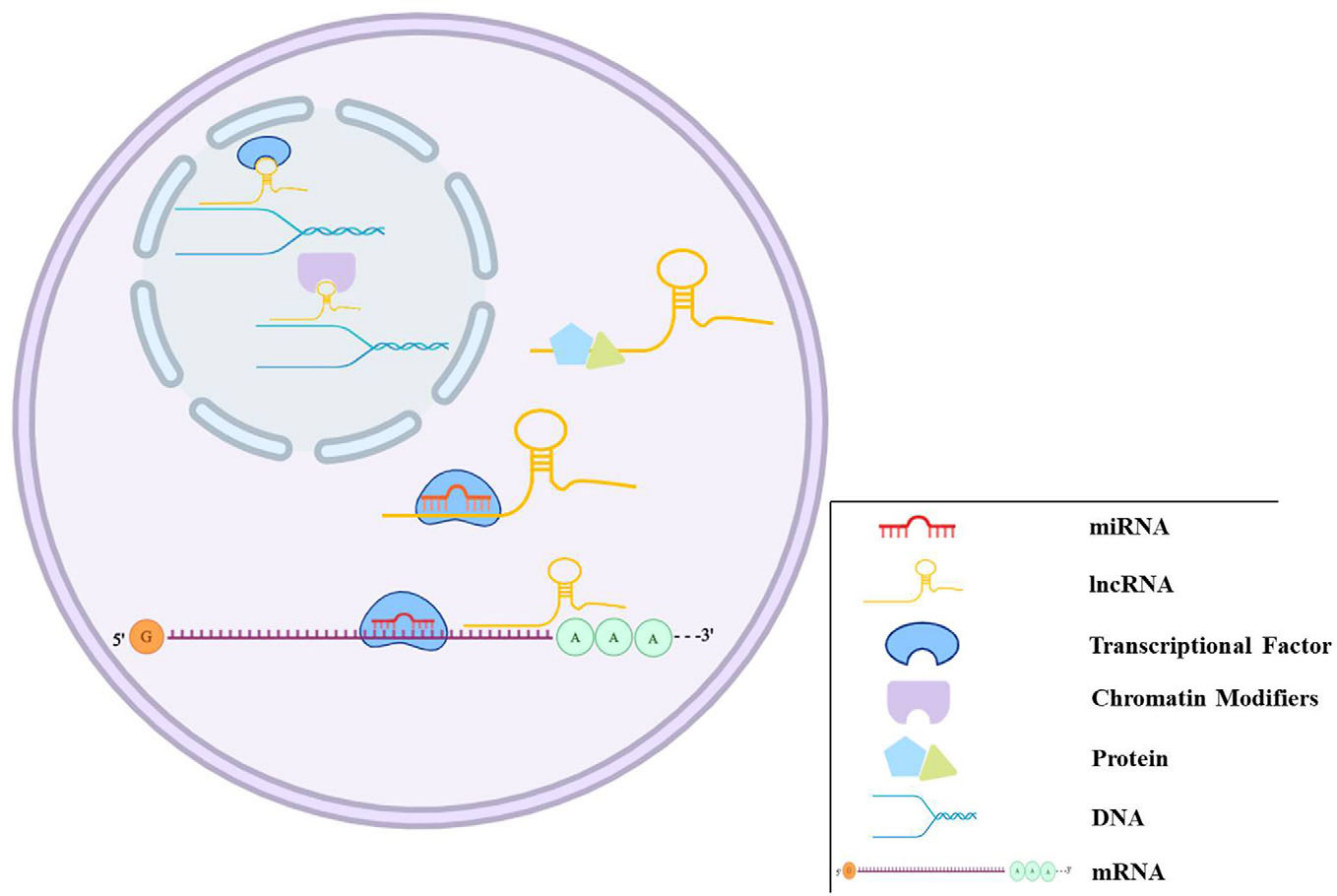
The regulation mechanisms of miRNAs and lncRNAs. miRNAs can bind to target genes to regulate gene expression. lncRNAs play regulation roles in different ways, such as transcriptional and post-transcriptional regulation, chromatin modification, miRNAs sponge, and binding to proteins.

Over the last few years, the noncoding RNAs have attracted significant interest in the scientific community. High-throughput RNA-sequencing analysis of the genome has shown that 70% of the human genome is transcribed, but only 2% code for proteins (Keiner et al., 2013). Regulatory noncoding RNAs are important members of the noncoding RNA family, including microRNAs (miRNAs), long noncoding RNAs (lncRNAs), and circular RNAs (circRNAs). (The noncoding RNA family is shown in **Figure 1**). MiRNAs are a class of noncoding RNAs approximately 22 nucleotides in length and act as negative regulators of gene expression by binding to the 3’ UTR of mRNAs (Khalil et al., 2009). lncRNAs are more than 200 nucleotides in length and rarely exhibit any protein-coding potential. MiRNAs are distributed in both the nucleus and cytoplasm, where they control chromosome modification and inhibit transcription. LncRNAs are distributed in the cytoplasm, where they target mRNAs and regulate the expression of target genes at the transcription level (Czubryt et al., 2003; Backs et al., 2006) or inhibit the function of miRNAs through combined targeting (Vega et al., 2004). The primary mechanisms of miRNAs and lncRNAs are summarized in **Figure 2**. As the regulatory noncoding RNAs, miRNAs and lncRNAs are shown to play significant roles in regulating CVDs (Wang et al., 2014; Viereck et al., 2016; Chu et al., 2018; Liu et al., 2018), also including HF (Gomes et al., 2020). Additionally, certain noncoding RNAs are stable in the blood, and their altered expression represents various disease states, indicating that they may function as significant CVD biomarkers, including HF. The relative research findings are summarized and shown in **Tables 1** and **2**.

**Table 1:** miRNAs as biomarkers in HF.

miRNA ID	Change in expression	Sample matrix	Study description	Control group	Main finding	Reference
miR-21-5p miR-30a-3p miR-30a-5p miR-155-5p miR-216a miR-217	↑ ↑ ↑ ↑ ↑ ↑	Plasma	HF(n=62)	Healthy control (n=62)	By RefFinder and PCR analysis, the six miRNAs were all upregulated compared with miR-39 as a reference. The ROC analysis by MedCalc showed that all AUC values are greater than 0.5, demonstrating that the detection method was effective. Correlation analysis indicated that the six miRNAs could be combined in two or three or more combinations to become a new biomarker for HF.	(Ding et al., 2020)
miR-150-5p	↓	Blood	UVH (n=48)	Healthy control (n=32)	With the help of UVH, miR-150-5p was the one of three most significant predictors of overt HF by ROC analysis (AUC 0.905, 95% CI 0.779-1.000; p=0.001).	(Abu-Halima et al., 2019)
miR-423-5p	↑	Plasma	HF (n=12) HF (n=30) Non-HF Dyspnea (n=20)	Healthy control (n=12) Healthy control (n=39)	A miRNA array and following real-time PCR were performed in two groups. It was specifically enriched in HF and AUC=0.91 (*P*<0.01). The ROC curve analysis showed miR-425 -5p to be a diagnostic predictor of HF.	(Tijsen et al., 2010)
miR-129-5p	↓	Plasma	UVH and HF (n=71)	NA	miR-129-5p is a sensitive and specific biomarker for heart failure in UVH disease independent of ventricular morphology or stage of palliation.	(Ramachandran et al., 2017)
miR-22 miR-92b miR-320a miR-423-5p	↑ ↑ ↑ ↑	Serum	Stable chronic systolic HF (n=30)	Healthy control (n=30)	The four miRNAs in HF group were >1.2-fold higher than those in controls, and all AUC > 0.76. The panel of four miRNAs indicate HF with a sensitivity and specificity of 90% and have a significant association with clinical prognostic parameters such as serum natriuretic peptide levels, a wide QRS et al.	(Goren et al., 2012)
miR-21	↑	Serum	HF, LVEF<50%, history of HF>6 months (n=80)	LVEF≥50%, no symptoms (n=40)	Both miR-21-CS and –PV have high levels of sensitivity and specificity for diagnosing HF. Both have correlation with prognosis, and miR-21-CS is efficient in predicting re-hospitalization for HF.	(Zhang et al., 2017)

ROC, Receiver operating characteristic; AUC, Area under curve; UVH, uni-ventricular heart; CI, Confidence interval; LVEF, Left ventricular ejection fraction; CS, Coronary sinus; PV, Peripheral vein; ↑: increase, ↓: decrease.

**Table 2:** lncRNAs as biomarkers in HF.

lncRNA ID	Change in expression	Sample matrix	Study description	Control group	Main finding	Reference
PVT1	↑	Serum	CHF (n=92)	Healthy control (n=60)	PVT1 was upregulated, and its target, miR-190a-5p, was downregulated in CHF patients. Although the both could become independent diagnostic biomarkers of CHF, the combination of PVT1 and miR-150a-5p showed better diagnostic accuracy.	(Sun B. et al., 2020)
LIPCAR	↓ → ↑	Plasma	Ischemic HF (n=164)	Nonischemic HF (n=180)	The expression of LIPCAR: early after MI ↓, later after MI ↑, CHF ↑↑.The level of LIPCR improved the prediction of cardiovascular death, including HF.	(Kumarswamy et al., 2014)
H19	↑	Plasma	CAD (n=300)	Healthy control (n=180)	The level of H19 was increased in CAD patients with HF, and AUC=0.63. Multivariate logistic regression analyses indicate that H19 was independent predictor for CAD.	(Gomez et al., 2018)
NRON MHRT	↑ ↑	Plasma	HF(n=72)	Non-HF control (n=60)	The area under the ROC curve was 0.865 for NRON and 0.702 for MHRT. NRON was negatively correlated with HDL and positively correlated with LDH; MHRT was positively correlated with AST and LDH.	(Xuan et al., 2017)
ANRIL HOTAIR TUSC7	↑ ↓ ↓	LV heart tissue/PBMCs	HF (n=54)	Healthy control (n=52)	RT-qPCR was used to detect the expression of lncRNA in LV heart tissue and PBMCs from non-end-stage, end-stage HF patients, and healthy individuals; the expression changes were similar in the two samples, suggesting a potential as disease biomarker.	(Greco et al., 2016)
UCA1	↑	Plasma	CHF (n=64)	Healthy control (n=64)	CHF patients with higher UCA1 levels had a lower survival rate compared with those with a lower level. UCA1 diagnosed CHF with a diagnostic power of 0.89 and a sensitivity and specificity of 100% and 76.12% (*P*<0.05).	(Yu et al., 2017)

CHF, Chronic heart failure; CAD, Coronary artery disease; MI, Myocardial infarction; AUC, Area under curve; HDL, High-density lipoprotein; LDH, Lactate dehydrogenase; AST, Aspartate aminotransferase; LV, Left ventricular; PBMCs, Peripheral blood mononuclear cells; ↑: increase, ↓: decrease, →: change.

## circRNAs in HF

**Figure 3:**
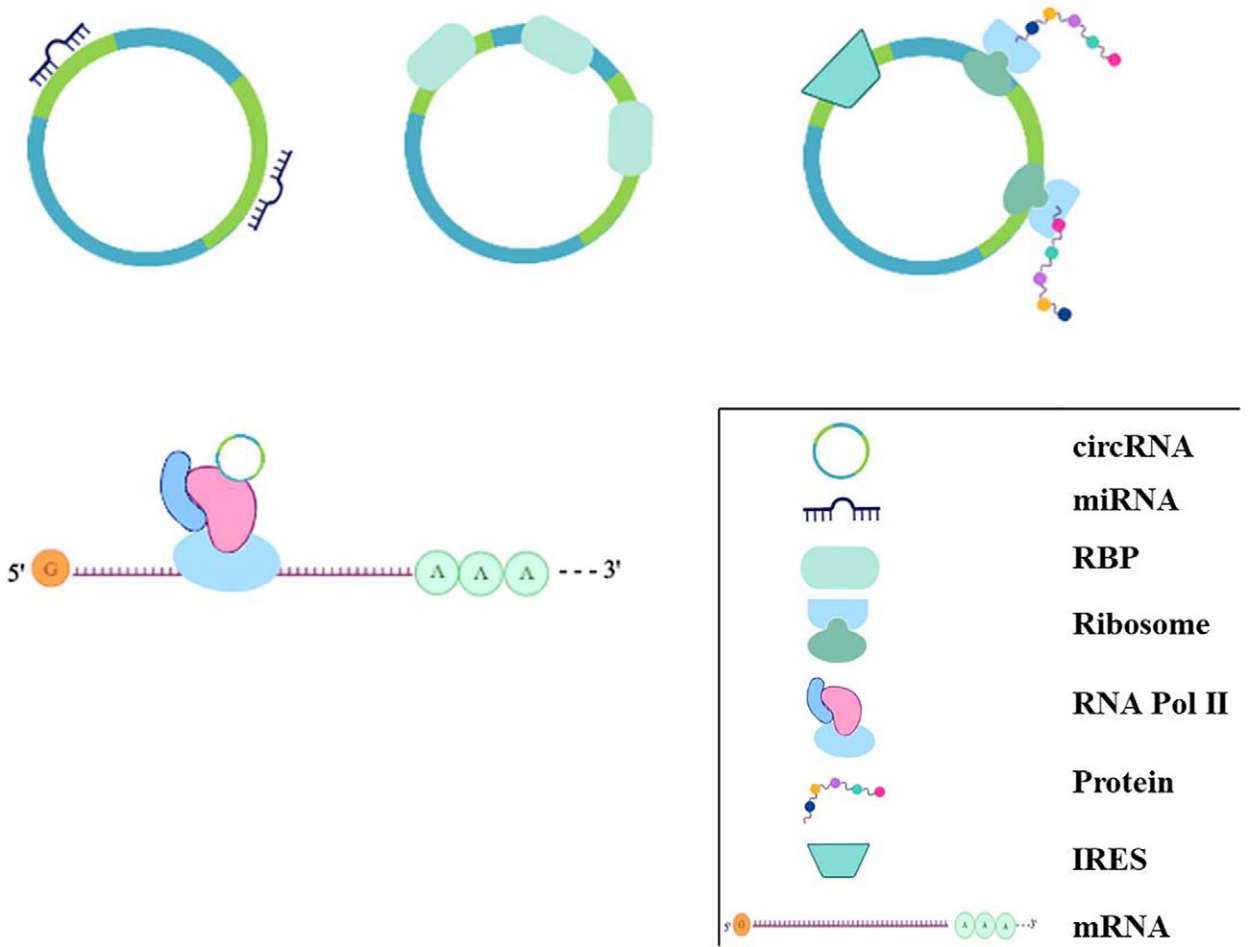
The regulation mechanisms of circRNAs. circRNAs can bind to miRNAs acting as miRNA sponges; some circRNAs can bind to protein, especially RBP, acting as sponge or decoy; some can regulate transcriptionally through interacting with RNA Pol II, or some circRNAs even have the potential role of coding for proteins by internal ribosome entry site.

Over the past few years, research on noncoding RNAs implicated in the pathogenesis of HF has rapidly progressed and is still ongoing. Similar to miRNAs and lncRNAs, circRNAs are a class of noncoding RNAs, but unlike linear RNAs, circRNAs form a closed continuous loop, signifying that the structure has neither 5’ caps nor 3’ tails (Chen and Yang, 2015). circRNAs are primarily produced by exons or introns, and reverse complements or RNA-binding proteins (RBPs) are required for its “life-forms” (Liang and Wilusz, 2014; Vicens and Westhof, 2014; Petkovic and Muller, 2015). As functional noncoding RNAs, the finding of circRNAs has undergone a similar process of those in miRNAs and lncRNAs, from molecular flukes or products of aberrant RNA splicing to research hot spots on the subject of RNA. Although the structure, biogenesis, and functioning of circRNAs need to be studied further, it is still possible to deduce their pathological and physiological functions. CircRNAs function as sponges for miRNAs to regulate the expression of target genes (Hansen et al., 2013; Memczak et al., 2013) and directly regulate transcription with RNA Pol II or protein coding (Ashwal-Fluss et al., 2014; Chen, 2016; Yang et al., 2017). The key mechanisms of circRNAs are summarized in **Figure 3**. Increasing evidence has revealed the functions of naturally occurring or regulatory circRNAs in the development of cardiovascular diseases, including cardiac hypertrophy, acute myocardial infarction, cardiac cell senescence, and diabetic cardiomyopathy (Wang et al., 2016; Du et al., 2017; Xia and Song, 2020; Zhang Q. et al., 2020).

CircRNAs involved in HF are widely discussed. (This information is described in **Table 3**). The circRNA-miRNA-mRNA axis is an important regulatory mechanism in HF. CircSlc8a1, a circRNA that is highly expressed in cardiomyocytes (Tan et al., 2017) and highly conserved across vertebrate species, is proven to regulate HF as an endogenous miRNA sponge (Lim et al., 2019). RNA pull-down and qPCR analysis shows miR-133 and miR-1 were detected to be circSlc8a1-bound. Next, a luciferase assay and biotin-based pull-down assay shows that there was a robust endogenous interaction between circSlc8a1 with miR-133a and not miR-1 although the circSlc8a1 sequence contains a putative binding site for miR-1. Forced circSlc8a1 expression in mouse heart *via* an AAV9-based vector system would result in HF *via* regulating the expression of serum response factor (Srf), connective tissue growth factor (CTGF), β1-adrenergic receptor (β1-AR) *via* miR-133a or adenylate cyclase 6 (Adcy6) directly. Interestingly, the expression of Adcy6 is also affected by circ-HPIK3 (Deng et al., 2019). Overactivation of β-AR can improve heart function temporarily *via* regulating Ca^2+^ load and transient (Morgan et al., 1990; Reiken et al., 2003) but will be a risk factor of HF long term. Deng et al. found that adrenaline could induce the expression of circ-HPIK3 *via* transcription factor cAMP responsive element-binding protein 1 (CREB1). Circ-HPIK3 would impair the function of heart *via* increasing Ca^2+^ concentration by the circ-HPIK3-miR-17-3p-Adcy6 axis in the long run and might be a therapeutic target of HF.

**Table 3:** circRNAs as regulatory noncoding RNAs in HF.

circRNA ID	Change in expression	Disease model	Model/species	Implication in HF	Main finding	Reference
circSlc8a1	↑	Hypertrophy	mouse	Upregulation aggravates HF	CircSlc8a1 induced HF *via* regulating the expression of Srf, CTGF, β1-AR *via* sponging miR-133a or Adcy6 directly.	(Lim et al., 2019)
HRCR	↓	Hypertrophy	mouse	Upregulation alleviates hypertrophy	HRCR attenuated cardiac hypertrophy by targeting miR-223.	(Wang et al., 2016)
circRNA_000203	↑	Hypertrophy	mouse	Upregulation aggravates hypertrophy	CircRNA_000203 could sponge miR-26b-5p and miR-140-3p, abolish their synergistic inhibition of Gata4, resulting in aggravating hypertrophy.	(Li et al., 2020)
circ-HPIK3	↑	MI	mouse	Upregulation aggravates MI	The expression of circ-HPIK3 was regulated by adrenaline *via* CREB1, circ-HPIK3-miR-17-3p- Adcy6 axis played roles in regulating heart function.	(Deng et al., 2019)
circNfix	↑	MI	mouse rat	Downregulation alleviates MI	The expression of circNfix was regulated by Meis1, and circNfix reinforced the interaction of YBX1 and NEDD4L to decrease YBX1, and regulated Gsk3β signaling by miR-214.	(Huang et al., 2019)
CDYL	↓	MI	mouse	Upregulation alleviates MI	CircRNA CDYL promoted proliferation of cardiomyocytes after MI through miR-4793-5p/APP pathway.	(Zhang M. et al., 2020)
circFndc3b	↓	MI	mouse human	Upregulation alleviates MI	CircFndc3b attenuated cardiomyocyte apoptosis *via* interaction with FUS to regulate VEGF-A.	(Garikipati et al., 2019)
ACAP2	↑	MI	rat	Upregulation aggravates MI	CircRNA ACAP2 had better stability and resistance to RNase R. ACAP2 promoted the apoptosis of cardiomyocytes through binding to miR-29.	(Liu et al., 2020)
circRNA 010567	↑	MI	rat	Upregulation aggravates MI	Decreased the expression of circRNA 010567 could improve the cardiac function, alleviated the myocardial fibrosis by inhibiting TGF-β1 signaling pathway.	(Bai et al., 2020)
circ_LAS1L	↓	MI	human	Upregulation alleviates MI	30 AMI patients and 30 healthy volunteers were enrolled in this study. Circ_LAS1L inhibited cardiac fibroblasts proliferation by sponging miR-125b to increase the expression of SFRP5.	(Sun L. Y. et al., 2020)

Srf, Serum response factor; CTGF, Connective tissue growth factor; β1-AR, β1-adrenergic receptor; Adcy6, Adenylate cyclase 6; Gata4, Gata binding protein 4; CREB1, cAMP responsive element-binding protein 1; YBX1, Y-box bingding protein 1; NEDD4L, an E3 ubiquitin ligase; APP, Amyloid β precursor protein; FUS, FUS RNA binding protein, VEGF-A, Vascular endothelial growth factor-A; SFRP5, Secreted frizzled-related protein 5; ↑: increase, ↓: decrease.

CircRNAs involved in HF have been widely discussed because cardiac hypertrophy contributes to HF. Heart-related circRNAs (circRNAs HRCR) are reported to attenuate cardiac hypertrophy by targeting miR-223. The expression of circRNA HRCR is reduced in thoracic aortic constriction (TAC) and isoproterenol-induced cardiac hypertrophy mouse models (Wang et al., 2016). The expression of circRNA_000203 was increasing in *in vitro* and *in vivo* models of cardiac hypertrophy (Li et al., 2020). Overexpression of circRNA_000203 both *in vitro* by transfection recombinant adenovirus into neonatal mouse ventricular cardiomyocytes (NMVCs) and *in vivo* transgenically would induce a hypertrophy phenotype. CircRNA_000203 could sponge miR-26b-5p and miR-140-3p and abolish the synergistic inhibition of Gata4, a pro-hypertrophy factor. Furthermore, NF-κB signaling also took part in cardiac hypertrophy regulated by circRNA_000203.

In addition, ischemic-induced cardiac remodeling is also an important promoter of HF. Myocardial infarction (MI) is an ischemic cardiomyopathy caused by insufficient coronary blood supply and induces necrosis and apoptosis of cardiomyocytes. The remaining cardiomyocytes cannot achieve self-repair and are forced to undergo compensatory hypertrophy. At the same time, the proliferation of fibroblasts is activated, resulting in ventricular remodeling and myocardial fibrosis. A long-term cardiac workload will eventually lead to HF (Pouleur et al., 2010). It is reported that the transcription factor Meis1 has a regulatory effect on the regeneration of cardiomyocytes. A recent study showed that Meis1 bounded to the super-enhancer at the circNfix locus and increased its expression (Huang et al., 2019). However, loss of super-enhancer-regulated circNfix could promote cardiac regeneration after MI. This study may indicate that circNfix may be a key regulator for improving the prognosis after MI and inhibiting the progression of HF. This also further explains the proliferation ability of adult mice being reactivated after Meis1 knockout (Mahmoud et al., 2013). CDYL is another circRNA reported to modulate cardiac regeneration after acute MI (Zhang M. et al., 2020). Overexpression of circRNA CDYL could promote proliferation of cardiomyocytes *in vitro* through sponging miR-4793-5p. These circRNAs attenuate the development of HF by regulating the proliferation of cardiomyocytes after MI.

CircRNA circFndc3b was significantly downregulated in post-MI hearts and also decreased in cardiac tissues of ischemic cardiomyopathy patients. AAV-9-mediated overexpression of circFndc3b reduced cardiomyocyte apoptosis and improved angiogenesis and contractile function in a rat model of MI *via* interaction with RNA binding protein FUS (Garikipati et al., 2019). Similarly, circRNA ACAP2 exacerbated the development of HF *via* promoting the apoptosis of cardiomyocytes by targeting to miR-29 (Liu et al., 2020). The two circRNAs modulate the progression of HF by regulating the cardiomyocyte apoptosis after MI.

In addition, preventing ventricular remodeling is an effective way to inhibit HF. MI-induced myocardial fibrosis (MF) is an important mechanism to induce ventricular remodeling. Silencing circRNA 010567 in the rat model of acute MI established that using ligation of the left anterior descending coronary artery would improve cardiac function, higher ejection fraction, and fractional shortening and alleviate MF through the TGF-β1 signaling pathway (Bai et al., 2020). A cardioprotective circRNA, circ_LAS1L, was found markedly downregulated in acute MI (Sun L. Y. et al., 2020). Overexpression of circ_LAS1L could inhibit cardiac fibroblast proliferation and migration and promote apoptosis. Mechanistically, circ_LAS1L sponged miR-125b to upregulate the expression of secreted frizzled-related protein 5 (SFRP5) and inhibit the expression of α-SMA, collagen I, and collagen III to play an important role in the process of MF.

These findings demonstrate that circRNAs have potential regulatory functions in HF and inhibit symptoms that can induce HF, including hypertrophy, cardiomyocyte apoptosis, and ventricular remodeling. Interestingly, circRNAs have been detected in the bloodstream (Vausort et al., 2016), signifying the possibility of constituting a reservoir of novel biomarkers. In addition, due to their putative ability to aid in the advancement of personalized health care of patients with HF and their presence in circulation, circRNAs can be used as potential diagnostic tools.

## circRNAs as biomarkers of HF

Similar to miRNAs and lncRNAs, circRNAs also have the potential of being used as biomarkers of HF and can even be more effective than miRNAs and lncRNAs. First, circRNAs have excellent stability associated with their circularized structure, which protects them from endonuclease activities (Memczak et al., 2015). Therefore, the half-life of circRNAs in cells is longer than that of other lncRNAs that are released by extracellular vesicles (Alhasan et al., 2016). Second, circRNAs are widely distributed and can be detected in blood, plasma, and extracellular vesicles (Koh et al., 2014; Vausort et al., 2016). Third, circRNAs are present in large amounts in the blood (Memczak et al., 2015), exceeding the corresponding linear mRNAs by more than 10-fold (Jeck et al., 2013). Fourth, RNA-sequencing technology has identified hundreds of cell-specific circRNAs in human and mouse cells and tissues, providing more candidates for the selection of disease biomarkers (Salzman et al., 2013). CircRNAs have been described as potential biomarkers for atherosclerotic cardiovascular disease risk, depressive disorder, aging, and cancer despite inadequate evidence of their functions in HF (Burd et al., 2010; Westholm et al., 2014; Bahn et al., 2015; Li et al., 2015; Chen et al., 2017). Therefore, there is evidence that circRNAs are potential biomarkers for HF.

There is increasing evidence indicating that the expression of specific circRNAs is dysregulated in cardiovascular disease. For example, hsa_circ_01224644 has been reported as a potential biomarker of coronary artery disease due to its upregulation during the progression of the disease (Zhao et al., 2017). In addition, the circRNA has_circ_0001445 has been reported to enhance the identification of atherosclerotic coronary artery disease (Vilades et al., 2020). RNA sequencing analysis was performed on human failing hearts and TAC mice in a previous study (Werfel et al., 2016). The study detected three circRNAs that were significantly altered; expressions of m005501 and m005492 were upregulated, whereas m005505 expression was downregulated. People have put forth that two independent studies have shown that two circRNAs were identified as differentially expressed when comparing young and aged hearts (Du et al., 2017; Zeng et al., 2017). Circ-FOXO3 was significantly expressed in aged human and mouse hearts, and circ-Amotl1 was highly expressed in neonatal human heart tissue. Whether these two circRNAs can be put together to indicate the degree of HF is worthy of our study. Consequently, circRNAs are appropriate for the diagnosis of cardiovascular diseases and are potential biomarkers of HF. The changes of circRNAs as biomarkers of HF are summarized in **Table 4**.

**Table 4:** circRNAs as biomarkers in HF.

circRNA ID	Change in expression	Sample matrix	Study description	Control group	Main finding	Reference
m005501 m005492 m005505	↑ ↑ ↓	Heart tissue	Adult rat (n=3)	Neonatal rat (n=3)	RNA sequencing analysis was performed on heart tissue of HF model, including human, rat, and mouse. These three circRNAs had a significant change in expression, indicating they might have a directive function of HF.	(Werfel et al., 2016)
			TAC mice (n=2)	Sham mice (n=3)		
			HF patient (n=2)	Non-failing heart (n=2)		
circ-FOXO3	↑	Heart tissue	Human >50 years (n=11)	Human <50 years (n=9)	The expression of circ-FOXO3 was higher in aged hearts. Inversely, circ-Amotl1 was highly expressed in neonatal hearts. These two circRNAs can be put together to indicate the degree of HF	(Du et al., 2017)
		CMs	CMs isolated from neonatal mice	CMs isolated from 12 week mice		
circ-Amotl1	↓	Heart tissue	The population enrolled in this study was aged from younger than 1 years to 76 years old			(Zeng et al., 2017)
MICRA	↓	Blood	ST-elevation MI (n=270) non ST-elevation MI (n=139)	healthy volunteers (n=86)	Evaluating the subjects’ expression of MICRA and heart condition through left ventricular function, ejection fraction, demographic, and clinical variables, circRNA MICRA could be used as a biomarker of HF. In addition, some other circRNAs were also detected; these circRNAs might play roles in the diagnosis of HF combined with MICRA.	(Yvan et al., 2017)
CFNDC3B cBPTF cEXOC6B cLAMA2-2 cPLCEI cPRDM5	↑ ↑ ↑ ↑ ↑ ↑	LV tissue	DCM (n=26) ICM (n=17)	Control (n=23)	The 6 circRNAs were identified with reproducible associations with HF and had a significant expression differential and stability, and therefore, key biomarkers for the diagnosis of HF and/or predicting the clinical evolution of HF in a patient.	(Yvan and Lu, 2018)
hsa_circ_0062960	↑	Plasma	Chronic stable HF (n=30)	Control (n=30)	The expression of hsa_circ_0062960 was highly correlated with BNP. GO and KEGG pathway analyses shown the expression to also be related to platelet activity.	(Sun Y. et al., 2020)
DNAJC6, TMEM56 MBOAT2	↓ ↓ ↓	Serum	HNCM (n=33) HOCM (n=31)	Control (n=53)	CircRNAs TMEM56 and DNAJC6 were negatively correlated with echocardiographic parameters for HOCM and could be used as indicators of disease severity in patients with HOCM.	(Sonnenschein et al., 2019)
hsa_circ_0001445	↓	Plasma	Stable CAD (n=200)		The stability of hsa_circ_0001445 was detected in room temperature, 4°C, freeze/thaw cycles and hemolysis. It will also improve the accuracy of diagnose CAD.	(Vilades et al., 2020)
has_circ_0005540	↑	Plasma	CAD (n=108)	Non-CAD (n=89)	An exoRNwasy Serum/Plasma Midi kit was used to isolate total exosome RNA from plasma. Has_circ_0005540 was selected from 355 circRNAs, which had a remarkably fold change and associated with CAD.	(Wu et al., 2020)
has_circ_0097435	↑	Plasma	HF (n=45)	Healthy volunteer (n=44)	The expression of exosmal has_circ_0097435 was increased in HF patients. In vivo, overexpression of has_circ_0097435 could induce cardiomyocyte apoptosis, and silencing has_circ_0097435 inhibited apoptosis. It will also play roles in HF by sponging multiple miRNAs.	(Han et al., 2020)

CMs, Cardiomyocytes; DCM, Dilated cardiomyopathy; ICM, Ischemic cardiomyopathy; GO, Gene Ontology; KEGG, Kyoto Encyclopedia of Genes and Genomes; HNCM, Non-obstructive hypertrophic cardiomyopathy; HOCM, Obstructive hypertrophic cardiomyopathy; CAD, Coronary artery disease; ↑: increase, ↓: decrease.

A previous study demonstrated that the expression of circRNA referred to as MI-associated circular RNA (MICRA) is reduced in patients with acute MI compared with healthy people and may act as a predictor of the risk of left ventricular dysfunction after acute MI. MICRA is also downregulated in patients with decreased ejection fraction, suggesting its potential role as a HF biomarker (Vausort et al., 2016). Recently, a patent file (Google patent: WO2017046203A1) demonstrated that the expression of MICRA, circ_0000005, circ_0000673, circ_0000585, circ_0000816, circ_0000817, circ_0000917, circ_0001423, circ_0000540, circ_0001844, circ_0000994, and circNPPA is out of tune in HF. A total of 496 people were enrolled in this trial, including healthy volunteers (*n*=86), ST-elevation MI (*n*=270), and non-ST-elevation MI (*n*=139). The expression of MICRA in healthy volunteers and MI patients are significantly different, but there were none in ST-elevation or non-ST-elevation MI. In this trial, the expression levels of MICRA were measured by quantitative PCR. Beyond this, left ventricular function, ejection fraction and demographic and clinical variables were assessed at a 4-month follow-up to evaluate the subjects’ heart condition. Furthermore, the patient also maintained the use of the listed circRNAs in the diagnosis of HF, indicating that circRNA MICRAs can be used as biomarkers for HF (Bayoumi et al., 2018). In addition, this team also identified novel HF-associated circRNAs as biomarkers for HF (Google patent: WO2018220185A1). These circRNAs, including cFNDC3B, cBPTF, cEXOC6B, cLAMA2-2, cPLCEI, and cPRDM5 were differentially expressed between subjects with failing hearts and subjects with nonfailing hearts. Therefore, these novel circRNAs most likely have a function in HF and further can be used as biomarkers for diagnosis of HF for the prediction of the clinical evolution of HF, such as the prediction of the development of cardiac decompensation. Similarly, in another study, hsa_circ_0062960 exhibited a higher expression in patients with HF, and the area under the curve (AUC) for HF diagnosis was 0.838 (*p*<0.0001). Notably, correlation analysis shows that the expression of hsa_circ_0062960 is highly positively correlated with the serum level of BNP, signifying its potential role as a biomarker of HF (Sun Y. et al., 2020).

There were three circRNAs identified as potential biomarkers for the hypertrophic cardiomyopathy (HCM) (Sonnenschein et al., 2019). Serum expression levels of the three circRNAs, DNAJC6, TMEM56, and MBOAT2, were identified in blood samples from 64 patients with HCM and 53 healthy controls and were both downregulated. Abundance of circRNAs was correlated to relevant clinical parameters, and the result showed that the three circRNAs could distinguish between healthy and HCM patients. In addition, TMEM56 and DNAJC6 could also serve as indicators of disease severity in patients with HCM. Of note, circRNA hsa_circ_0001445 exhibited remarkable stability tested under different experimental conditions in samples from 5 healthy blood donor volunteers (Vilades et al., 2020). In a real-world clinical practice setting containing 200 consecutive patients with suspected stable coronary artery disease (CAD), the classification of patients was much more accurate with the incorporation of has_circ_0001445 into a base clinical model composed of conventional cardiovascular risk factors, thus supporting its role as a biomarker of CAD.

Circulating noncoding RNAs occur in serum in 2 forms: free nucleic acids and nucleic acids contained in exosomes. Noncoding RNAs isolated from exosomes are tested at higher sensitivities than those in the serum (Gallo et al., 2012). Exosomes are extracellular vesicles (EVs) derived from multivesicular bodies (MVBs) and are distinguished from microvesicles (MVs) and apoptotic bodies by size and biogenesis (Maas et al., 2017). Exosomes can function as biomarkers of HF predominantly through their noncoding RNA cargos (Yuan et al., 2018). Exosomes encapsulate noncoding RNAs and then secrete them into peripheral blood, thus protecting them from enzymatic degradation. Cardiac ischemia/reperfusion (I/R) could increase the secretion of EVs; the following RNA-seq of EVs identified 185 significantly differentially expressed circRNAs, 119 downregulated and 66 upregulated, compared with the sham, suggesting that circRNAs in cardiac EVs might indicate development of I/R as biomarkers (Ge et al., 2019). Using circRNAs to improve the discriminatory power for CVD has been discussed (Wu et al., 2020). Exosmal circRNA expression levels in exosomes was analyzed from 3 plasma samples of CAD patients and 3 paired controls using RNA sequencing. CircRNA has_circ_0005540 was selected as a candidate biomarker for CVD from 164 upregulated circRNAs and 191 downregulated circRNAs because it had a significant differential expression (fold change > 4, *P* < 0.05) and associated with CVD (*P* < 0.0001). Has_circ_0005540 also showed a high discriminatory power for CVD in ROC analyses (AUC=0.853, 95% CI=0.799-0.906, *P* < 0.001). These results suggest that plasma exosomal has_circ_0005540 could be used as a promising biomarker of CVD. Exosomes extracted from peripheral blood samples of patients with HF and healthy patients (control group) were analyzed using next-generation sequencing, and the results indicate that the expression of has_circ_0097435 was upregulated in patients with HF. The high expression of hsa_circ_0097435 in exosomes demonstrates that most hsa_circ_0097435 are encapsulated in the exosomes, confirming that hsa_circ_0097435 is a potential biomarker of HF (Han et al., 2020).

## Limitations

That circRNAs can become HF biomarkers is not random. For example, a recent study examining 953 patients with chronic and symptomatic HF revealed a positive correlation between miR-132 expression levels and the critical state of HF and HF hospitalizations (Masson et al., 2018). In patients with acute decompensated HF, the reduced levels of ncRNAs are attributed to fluid overload. This also attracts an interesting opinion that tracing the origin of circulating ncRNAs in HF is important. CircRNAs act as attractive therapeutic targets and biomarkers for HF because of their peculiarity. The identification of circRNA profiles in HF may result in new diagnostic tools. CircRNAs may also have an added advantage over other existing methods and biomarkers by providing additional information to guide clinical decisions. Therefore, there is convincing evidence on the potential of circRNAs as biomarkers of HF in clinical applications. However additional research should be conducted to resolve the hurdles and promote the application of circRNAs as biomarkers for HF. The use of circRNAs as biomarkers for HF is partially limited by both technical and nontechnical barriers.

Accuracy is a crucial determinant in the use of circRNAs as biomarkers of HF. Studies have demonstrated that a certain miRNA was downregulated in patients with acute HF but not in chronic HF patients (Ovchinnikova et al., 2016). This implies that the noncoding RNA biomarkers should be used in hierarchical prediction to ensure accuracy and effectiveness. Confounding factors, such as age, sex, cardiovascular risk factors, and pharmacological treatments, among others, should also be considered in the prediction results (De Gonzalo-Calvo et al., 2019). In addition, the use of clusters of circRNAs rather than single circRNAs may improve accuracy.

Comparing and reproducing results from different studies, including study designs, materials, sample isolation, techniques, normalization strategies, and the influence of drugs and disease is challenging (Backes et al., 2015; Gomes et al., 2020). For example, most current studies do not have unified normalization strategies, and both endogenous and exogenous references vary. Isolation, purification, and characterization of exosomal circRNA biomarkers from MVBs, lipoproteins, and macromolecular complexes is still a major challenge despite the application of different techniques, including chromatography, centrifugation, precipitation, and affinity-isolation (Lawson et al., 2016; Takov et al., 2019). Fortunately, some studies on circRNA detection have already begun (Google patent: CN111378741A). Specfically, a molecular label containing a fluorescent reporter group and a quencher group were coupled on a nucleic acid probe targeting a circRNA. When the blood contained the circRNA to be detected, the circRNA was subjected to base complementary pairing, and the reaction liquid in the 5’ to 3’ exonuclease appraisal formed double-stranded nucleic acid group in order for the nucleotide sequence between the fluorescent reporter group and the quenching group on the nucleic acid probe to be cut; the fluorescent report group is dissociated from the nucleic acid probe targeting the circRNA into the reaction liquid, and then a fluorescent signal is emitted. Another limitation is that of the light wavelength in flow cytometry, which makes it difficult to distinguish exosomes from other EVs (Lawson et al., 2016). The use of fingerprint proteins, such as tetraspanins CD9, CD63, CD81, and CD82, may be a more effective method of isolating exosomes. However, this method can only distinguish exosomes from other EVs, and it is not possible to retrospectively determine which kind of cells derived them (Arraud et al., 2014; Kowal et al., 2016). Therefore, reliable methods and standardization strategies are required to enhance the translation of basic research into clinical practice. The present findings on the use of circRNAs as biomarkers of HF are very promising although further studies should be conducted to resolve the limitations of the currently used diagnostic markers.

## Conclusion and Future Prospects

The development of novel biomarkers for HF is of great significance considering the risks of HF and the limitations of existing diagnostic markers for HF. This article discusses the potential use of circRNAs as HF biomarkers. Despite the differences present in circRNAs examined in animal models or HF patients’ samples, assessment techniques that may influence their accuracy and the need for further research, the present findings strongly indicate that circRNAs can be used as effective predictive and diagnostic biomarkers of HF.

## Author Contributions

CS, MN, and BS wrote the manuscript. LC provided direction and edited the manuscript. All authors contributed to the article and approved the submitted version.

## Funding

This work was supported by grants from the Nanjing Medical University Science and Technology Development Foundation (2017NJMU059).

## Conflict of Interest

The authors declare that the research was conducted in the absence of any commercial or financial relationships that could be construed as a potential conflict of interest.
